# Advances for pediatricians in 2022: allergy, anesthesiology, cardiology, dermatology, endocrinology, gastroenterology, genetics, global health, infectious diseases, metabolism, neonatology, neurology, oncology, pulmonology

**DOI:** 10.1186/s13052-023-01522-8

**Published:** 2023-09-08

**Authors:** Carlo Caffarelli, Francesca Santamaria, Ettore Piro, Simona Basilicata, Lorenzo D’Antonio, Bertrand Tchana, Sergio Bernasconi, Giovanni Corsello

**Affiliations:** 1 Clinica Pediatrica, Department of Medicine and Surgery, Azienda Ospedaliera- Universitaria, University of Parma, Parma, Italy; 2 Department of Translational Medical Sciences, Federico II University, Naples, Italy; 3 Department of Sciences for Health Promotion and Mother and Child Care “G. D’Alessandro”, University of Palermo, Palermo, Italy; 4 Cardiologia Pediatrica, Azienda-Ospedaliero Universitaria, Parma, Italy; 5 Microbiome Research Hub, University of Parma, Parma, Italy

**Keywords:** Allergy, Anesthesiology, Cardiology, Dermatology, Endocrinology, Gastroenterology, Genetics, Global health, Infectious diseases, Metabolism, Neonatology, Neurology, Oncology, Pulmonology

## Abstract

The last year saw intensive efforts to advance knowledge in pediatric medicine. This review highlights important publications that have been issued in the Italian Journal of Pediatrics in 2022. We have chosen papers in the fields of allergy, anesthesiology, cardiology, dermatology, endocrinology, gastroenterology, genetics, global health, infectious diseases, metabolism, neonatology, neurology, oncology, pulmonology. Novel valuable developments in epidemiology, pathophysiology, prevention, diagnosis and treatment that can rapidly change the approach to diseases in childhood have been included and discussed.

## Background

The Italian Journal of Pediatrics in 2022 has published a high number of papers that can considerably ameliorate the management of several childhood illness. Among the most accessed papers of the past year, we present those thought to potentially have a significant impact. The selected articles highlight progresses in several fields: allergy, anesthesiology, cardiology, dermatology, endocrinology, gastroenterology, genetics, global health, metabolism, neonatology, neurology, oncology, pulmonology. Findings have been discussed and context of the studies has been provided with the aim of identifying changes in the clinical approach to the diseases.

### Allergy. 1‑ Food allergy; 2‑ Atopic eczema; 3‑ Asthma; 4- Hypersensitivity pneumonitis

#### 1- Food allergy

Food allergy is one of the most challenging field in pediatrics [1, 2]. It is common in developed countries resulting in an important problem for quality of life. Food allergy care has gone a long way. The Italian Society for Pediatric Gastroenterology Hepatology and Nutrition and the Italian Society for Pediatric Allergy and Immunology joint position paper [3] on the management of food allergy including activities and responsibilities of general practitioners, emergency departments and tertiary centers, such as food challenge [4, 5], has been published last year. The document provided information that will decrease delayed diagnosis, costs, and hazards for children with food allergy.

#### 2- Atopic eczema

Atopic eczema has several clinical phenotypes that needs a multidisciplinary approach [6]. The review by Galli et al. [7] reports current knowledge on treatment for moderate to severe cases. It has been highlighted the use of traditional treatments including trigger avoidance, emollients, bath, topical steroids, calcineurin inhibitors, immunosuppressive agents, anti-H1 antihistamines, phototherapy, education. Furthermore, it illustrates novel systemic drugs that acts on specific molecules involved in the immune mechanism that driven the pathogenetic pathways of AD, including biologics, janus kinase inhibitors and allergen specific immunotherapy [8]. An algorithm for clinical use of topical and systemic treatments is also provided.

#### 3- Asthma

Chronic airway inflammation leads to occurrence of symptoms in asthmatics [9–13]. Inhaled drugs are useful for asthma treatment since are effective and systemic adverse reactions are rare. In children with asthma, an inhaler both dry powder and pressurised metered-dose with and without the use of spacer should be used for delivering drugs to the lungs. So, a correct inhaler technique is one of the most important step for controlling asthma in children. McCrossan et al. [14] review data from several studies and describe the pros and cons of educational methods used to improve inhaler technique. They underline that researches in this field are warranted to identify the best outcome measure for inhaler technique in children. Furthermore, trials comparing interventions for teaching inhaler technique at different ages are lacking.

#### 4- Hypersensitivity pneumonitis

Mastrorilli et al. [15] address the ‘hot topic’ of hypersensitivity pneumonitis in children [16, 17]. They underline that on the basis of a suggestive history, the diagnosis relies on respiratory signs and symptoms, pulmonary function tests, characteristic HRCT nodular opacities, serum specific IgG levels, response to elimination of eliciting factor. Of note, aspergillus species are commonly considered the cause. However, other fungi such as Schizophyllum commune can be involved [18]. Bronchoscopy with bronchoalveolar lavage, and the provocation test may be helpful. Besides supportive treatment, the resolution generally takes place with trigger avoidance and systemic corticosteroids. Hydroxychloroquine, azithromycin, immunosuppressants, rituximab, nintedanib might be considered.

### Anesthesiology. 1‑ Sedation

#### 1- Sedation

In infant and children, the optimal treatment and management of diseases sometimes require high quality diagnostic procedures for which a prolonged inactive state are necessary and sedation needed. Some of the desire characteristics for the sedation regimen are rapid onset of action, predictable duration, rapid cessation of effects, few side effects and no need of rescue drug. Cossovel et al. [19] interestingly investigate the difference between two approach, combination of intranasal ketamine and intranasal dexmedetomidine compare with the the combination intranasal dexmedetomidine and oral midazolam. Compared with other studies in literature, the results look controversial, probably because of limits and confounding factors in the design of the studies [20–22]. Prospective studies are needed.

### Cardiology. 1‑ Cardiovascular risk

#### 1- Cardiovascular risk

Noncommunicable diseases are the current challenge for the sustainability of the health system in industrialized countries and among them cardiovascular diseases remain the main issue, being the leading cause of death, handicap, and health expenditure [23]. Atherosclerosis the matrix lesion, begins and can be detected in infancy, and its severity, extent and progression in childhood is related to exposure to risks factors such as dyslipidemia, hypertension, chronic kidney diseases, congenital heart and acquired heart diseases, cancer [24]. Several studies have shown an association between exposure to cardiovascular risk factor in childhood and subclinical atherosclerosis in adulthood but to date there is still limited evidence a lack of direct link between these factors with adult clinical cardiovascular diseases [25, 26].

### Dermatology. 1‑ Hemangioma; 2‑ Epidermolysis bullosa; 3‑ Congenital ichthyosis

#### 1- Hemangioma

Infantile hemangioma are benign tumors, quickly growing that are commonly located on the skin, but may also be in solid organ as brain or liver. They may go through gradual and spontaneous involution, but in some cases, depending on their location, they can lead to severe complications, ulceration, bleeding organ dysfunction or transient to permanent deformity which significantly impact patient and family quality of life. Propranolol has changed the history and the management of infantile hemangiomas [27], because of facility of administration and high success rate, becoming the first line treatment before glucocorticoids and surgery [28]. Although the mechanism underlying its effect is still unclear and controversies about side effects and potential safety issues [29]. Early and prompt diagnosis and treatment is essential [30].

#### 2- Epidermolysis bullosa

Epidermolysis bullosa is a chronic genodermatosis with different phenotypes and severity. Retrosi et al. [31] reported on treatment in 3 groups of patients: < 5 years, > 5–12 years and > 12–18 years. In the first group, the family should change lifestyle and burden of caring on quality of life is high. alliance between the health professional and parents can facilitate relationship between all family members [32]. In the second group, children have to be educated in accepting and understanding the disease and involved in the management to diminish complications. In the third group, adolescents have a deep knowledge of the disease. Denial of the disease and low adherence to treatment are common [33]. Authors found that 27 (13%) out of 215 patients died. Eighteen (8%) patients died because of sepsis and/or respiratory failure during the first months of life. Nine (5%) died for squamous cell carcinoma in adulthood [34].

#### 3- Congenital ichthyosis

Congenital ichthyosis is a rare disease. Mutations of more than 50 genes have been found. Genotype is often correlated with distinct form of the disease [35]. Serra et al. [36] found new variants of the ABCA12, KRT1 and ST14 genes. Mutations of ABCA12 gene is significantly associated with neonatal mortality [37]. Mutations of KRT1 gene can be hereditary or occur de novo. The epithelial barrier disruption is associated with infections and dyselectrolytemia. Mutations of ST14 gene are not clearly associated with a specific clinical picture. Authors highlight the importance of the molecular characterization to make an appropriate treatment, define the prognosis and the genetic counselling [38].

### Endocrinology. 1- Obesity; 2- Puberty; 3- Type 1 diabetes; 4- Hypothyroidism

#### 1- Obesity

Obesity is a chronic diseases with the greatest impact on public health with an increasing incidence over time. In adolescents, the reasons for obesity are various and among the most significant we might mention the wide prevalence of the obesity on a world scale with an increase also in low-income countries, the many complications that excess weight can cause at the level of various organs and systems and the high percentage of obese adolescents becoming obese adults. On the other hand, the therapeutic approach used in the last years that is based on the (difficult) attempt to change the lifestyle of obese adolescents has not yet given satisfactory results, especially in the long term. For this reason Nicolucci and Maffeis [39] dedicate an in-depth review both to the anti-obesity medications available (with particular attention to the glucagon-like peptide-1 analog) and to the bariatric surgery. The results obtained with the combination of lifestyle changes and new drugs seem encouraging but there is a need to know the long-term results on the efficacy in determining and maintaining weight loss, on the action on possible complications (cardiovascular), on the quality of life and the long term side effects [40]. Even with regards to bariatric surgery (currently, sleeve gastrectomy and “Rouxen-Y gastric bypass are two of the most popular bariatric procedures the results seem promising [41]. Bariatric surgery is considered very selectively for example by the American Society of Metabolic and Bariatric Surgery in adolescents with body mass index (BMI) ≥ 35 kg/m2 with major comorbidities or with a BMI ≥ 40 kg/m2 with minor comorbidities, However, as discussed by Martinelli et al. in a recent review “the use of bariatric surgery in adolescent patients is still limited, with significant disparities among countries. Reasons include ethical concerns raised by performing an irreversible and invasive procedure in adolescence, with potential life-long alterations” [42]. In conclusion, on the one hand there is an increasingly general agreement in treating adolescent obesity by deepening knowledge on the use of new generations of drugs and/or bariatric surgery combined with behavioral therapies that help change lifestyles and on the other hand, the undoubted difficulties of this path lead us to insist on programs for the global prevention of obesity. In childhood, drug dosing is often based on body weight. However pharmacokinetic is influenced by body weight [43]. This issue should be considered in obese children, but it has not been deeply studied [43]. So far, higher doses are required only for trimethoprim/sulfamethoxazole [44], among antibiotics, midazolam, inhaled corticosteroids. Data on dosing of low molecular weight heparins and vitamin D are unclear [45, 46]. So, further investigation on drug dosing are warranted in obese children.

#### 2- Puberty

Much attention has been given to early and delayed puberty. A worldwide growing decrease of puberty timing has been observed. Key features in the pathogenesis are nutrition in pregnancy, birth weight, dietary habits, physical activity, psychological factors, exposure to electromagnetic fields, endocrine-disruptors [47–49]. Ferrari et al. [50] showed in 577 girls that increased Z-score change from birth weight and higher BMI may predict earlier age at menarche and at thelarche. This underlines the role of modifiable factors in the onset of puberty.

Among delayed puberty causes, constitutional delay of growth and puberty, a variant of normal growth is frequent. It may induce psychological difficulties, poor school performance and perhaps lower final height [51–53]. So, therapeutic interventions have received interest in recent years. In adolescent > 14 years of age with psychological problems, a course of estradiol and progesterone in females or testosterone in males can be given. Importantly, despite clinical interest, the benefit of growth hormone on final height is uncertain [54].

#### 3- Type 1 diabetes

Type 1 diabetes is a disease that is heterogeneously growing in prevalence. Several studies have examined annual incidence that greatly varies from 0.1 to 100,000 person in China [55] to 62.3 per 100,000 person in Scandinavia [56] and from 19 to 45 per 100,000 person-years in different Italian regions. Passanisi et al. [57] found an incidence of 20.6/100,000 person/years with an increase of 43% between 2019 and 2021 during Coronavirus Disease (COVID-19) pandemics in Calabria region, Italy. This supports the view that Severe Acute Respiratory Syndrome Coronavirus-2 (SARS-CoV-2) infection may predispose to a diabetes onset [58]. They also found an increase of children with severe ketoacidosis that can be justified by shutting of many health services during the pandemics.

#### 4- Hypothyroidism

Menorrhagia is frequent after menarche onset. It is a challenging issue that often depend on anovulatory cycles due to the immaturity of the hypothalamic-pituitary-ovarian axis [59], but it can also be related to coagulopathy, pregnancy, medications, endocrine disorders, trauma, infection, cancer, structural pathologies, gastrointestinal bleeding [60]. Barbero et al. [61] delineated a girl with menorrhagia associated with short stature, bradycardia, dry skin, thinning hair. Hypothyroidism [62] was confirmed by ultrasound showing an expanded thyroid with non-homogeneous hypoechogenic structure and laboratory tests showing anemia, high thyrotropin releasing hormone level, low free thyroxin, high serum anti-thyroid peroxidase antibodies and antithyroglobulin antibodies levels. Levothyroxine treatment was associated with remission of symptoms and regulation of laboratory parameters. This case highlights that follow up of the pubertal development is of particular importance for timely identification of underlying disorders.

### **Gastroenterology. 1‑ Diet; 2‑ Cyclic vomiting syndrome**

#### 1- Diet

The effect of diet in the prevention and treatment of several conditions with gastrointestinal symptoms attracts constant attention. Pulvirenti et al. [63] summarized studies on diseases that are cured by eliminating the offending food or need an appropriate dietary management to prevent malnutrition. A causal role of food has been found in food allergy, including eosinophil esophagitis and regurgitation. In infancy, early introduction into the diet of foods such as peanuts, seems to reduce the likelihood of developing food allergy compared with delayed introduction [64]. In cystic fibrosis, the caloric intake should be 10–15% higher than in healthy children because of pancreatic insufficiency. So, diet should be supplemented with fat for energy requirements and pancreatic enzyme should be given [65]. Breast-feeding and diet rich in fruit, vegetables and n-3 fatty acids are associated with a lower risk of developing inflammatory bowel disease. A specific diet is necessary for inflammatory bowel disease because of the risk of malnutrition due to low caloric intake, enteropathy, metabolic conditions, or steroid treatment. In active Crohn’s disease, enteral or parental nutrition with polymeric formulas, is the first option for 6–8 weeks [66]. Short-bowel syndrome requires parental nutrition and gradually increase of enteral feeding that stimulates gastrointestinal functions. Breast milk or polymeric formulas are recommended in infants, oral or partially enteral solid foods in children.

#### 2- Cyclic vomiting syndrome

The Italian Society of Pediatric Gastroenterology, Hepatology and Nutrition and the Italian Society of Pediatric Neurology [67] provided a timely survey on diagnosis and treatment of cyclic vomiting syndrome in 51 tertiary centers [68]. They found a significant difference in diagnostic criteria between centers. Foods as triggers were more commonly identified by gastroenterologists and neuro-gastroenterologists than neurologist. Gastroenterologists more often diagnosed gastrointestinal diseases and checked ammonia and lactic acid levels. Besides supportive treatment, the most common medication given during vomiting, ondansetron followed by proton pump inhibitors, sedative, anti-H2 antihistamines, non steroidal antiinflammatories, steroids [69]. Prophylactic treatment [70] widely varied among centers. In about 50% of cases cyproheptadine was used, in 25% pizotifen, in 25% amitriptyline, in 15% mitochondrial supplements, in 10% anticonvulsants. Other drugs were aprepitant, propranolol, flunarizine, 5-hydroxytryptophan, proton pump inhibitors, paracetamol, magnesium supplementation. Probiotics have not been used [71]. Overall, there is a need for a consensus to standardize the management of cyclic vomiting syndrome.

### Genetics. 1- Cat Eye syndrome; 2- Deletion of chromosome; 3- Hao-Fountain syndrome

#### 1- Cat Eye syndrome

Cat Eye syndrome is due to duplication and inversion of part of chromosome 22 [72]. It is characterized by iris coloboma, anal atresia and ear anomalies in 40% of patients and it can have multiple malformations [72, 73]. Serra et al. [74] described an infant with cat eye syndrome associated with persistent hypoglycemia and subsequent cholestasis, abnormal midline structures, including aplasia of the anterior pituitary gland, abnormal stalk, and ectopic neurohypophysis with hypopituitarism with cortisol, thyroxine, growth hormone deficits, corpus callosum hypoplasia. This report emphasizes that clinical presentation of cat eye syndrome is variable [75]. So, the presence of additional malformations and/or abnormalities should always be considered.

#### 2- Deletion of chromosome

Contiguous gene syndromes are the result of a loss of multiple neighboring genes from a particular chromosomal segment. Contiguous gene syndromes have been identified in patients with various clinical features such as intellectual disability, developmental delay, and congenital anomalies [76]. Serra et al. [77] report on a female newborn with a 1p31.3p22.2 deletion of 20.7 Mb containing about ninety genes, inherited from the healthy mother with a smaller deletion (2.6 Mb) within the same centromeric region. Clinical phenotype included craniosynostosis, facial dysmorphisms with bilateral microphthalmia and coloboma, cleft palate, and a severe global developmental and growth delay [78]. In this report, in view of a better characterization of a genomic and phenotypic profile, the authors paid attention to about 20 out of the involved genes, considered loss of function intolerant according to their haploinsuficiency score [79].

A female term newborn with cardio-facio-cutaneous syndrome, a pathology belonging to RASopathies [80], a group of conditions caused by germline mutations in genes encoding components or regulators of the rat sarcoma/mitogen-activated protein kinase (RAS/MAPK) pathway was observed [81]. Fetal macrosomia and a prenatal diagnosis of omphalocele [82] suggested an array comparative genomic hybridization by amniocentesis that identified a 19p13.3 deletion including the MAP 2 K2 gene. Surgical correction of omphalocele took place on the second day of life. After discharge, at 1 month of age, she was readmitted for repeated episodes of vomiting, subtending a readily treated hypertrophic pyloric stenosis. The authors stress the need for integrated and individualized follow-up in all subjects with genetic syndromes, potentially carriers of abnormalities not evidentiable at birth [83].

#### 3- Hao-Fountain syndrome

Zampieri et al. [84] report a case of Hao-Fountain syndrome (HAFOUS) a haploinsufficient condition caused by variants in the Ubiquitin-Specific Peptidase 7 gene (USP7), located on chromosome 16p13.2. USP7 is the largest family of deubiquitinating enzymes. Recent studies have shown that USP7 [85], as well as characterizing a complex neurodevelopmental disorder, plays a vital role in the regulation of various physiological and pathological processes related to endocrine complications, predisposition to autoimmune diseases, immunological deficit and a wide variety of cancers. The patient was a 15-year-old female patient with a complex neurodevelopmental disorder involving both cognitive and neurologic areas in which whole exome sequencing diagnosed a large de novo heterozygous deletions affecting USP7. The description in this patient of isolated tubal torsion expands the HAFOUS spectrum phenotype [86, 87].

### Global Health. 1- Bullying; 2- Mobile phone; 3- Child abuse; 4- Burns; 5- Under-nutrition

#### 1- Bullying

A research carried out in Palermo, Italy, investigated the phenomenon of bullying [88], and includes an analysis of the data on bullying reported in the scientific literature [89, 90]. The authors have administered, to 22,455 school-aged children, attending one of the 58 secondary first-grade schools of Palermo during the school year 2017/18, a questionnaire of 30 items investigating the main areas related to bullying, such as physical, verbal and indirect bullying, observers of bullying, resiliency, and prosociality. Results showed an increase of bullying, reported in 2011 to be present in about 14% of young people, and involving up to 66% of young people in the area of verbal bullying. Bullying resulted more frequent in higher school classes, specifically in students attending third level classes in schools with lower socioeconomic index. The authors conclude that interventions and preventive measures in public health programs should be implemented to stem this phenomenon [91].

#### 2- Mobile phone dependence

Mobile phone dependence, considered a counterproductive use of mobile phone for interpersonal communication, information acquisition, self-expression, and leisure and entertainment, is a scourge of modern society that affects large segments of the youth population [92]. In this report the authors analyse the correlations between self-core evaluation (SCE) (a personality trait that encompasses an individual’s subconscious, fundamental evaluations about themselves, their own abilities, and their own control), mental state and cell phone addiction in a sample of high school students [93]. The results of the analysis, since SCE had a significant negative correlation with mobile phone dependence, show how SCE plays an important mediating role between mental health and mobile phone dependence [94, 95]. The authors conclude that improving high students’ SCE may be beneficial to improve the health status and reduce mobile phones dependence.

#### 3- Child abuse

Offidani et al. describe the process that led to the editing of the indicators of child abuse and the codification of three clinical pathways to apply in case of suspected child abuse at the Bambino Gesù Children’s Hospital [96]. Field work has identified three areas of assessment that constitute a new screening tool (ST) created in 2009 and used in clinical practice from 2010. The fourteen items included in the ST have been grouped in three clusters: anamnestic declarations or incongruences, carelessness/neglect and evident lesions at physical examination. All items of the ST are reported in the text. Adopting the ST from 2010 to 2020, the authors report improvement in diagnostic accuracy resulting in reduction of under-diagnosed cases and confirm that females are more likely to experience sexual abuse, while males are more likely to experience physical abuse. It would be useful to share these indicators with non-paediatric hospitals, that do not have the same experience in recognizing cases of child abuse [97–99].

#### 4- Burns

Tiruneh et al. [100] conducted this institutional-based cross-sectional study on children admitted for burns to South Gondar zone hospitals from 2015 to 2019. They used for data collection a checklist including sociodemographic characteristics, as nutritional status, comorbidity, availability of health insurance, medical history such as the place of the accident, the mode of occurrence, the timing of hospitalization, the degree of burn, the duration of hospitalization and the therapeutic measures taken [101, 102]. The mortality among burn victim children (8,5%) was higher than most of the studies conducted all over the world and similar to data from East Africa and Tanzania. No medical insurance, being malnourished, burnt by electrical and flame burn, having total body surface area burnt greater than 20%, and having poor clinical condition at admission increased mortality by four times compared to a good clinical condition. These results may suggest priority actions to reduce mortality and worst outcomes in children [103].

#### 5- Undernutrition

Undernutrition is a major public health issue especially in some countries of Africa and Asia [104]. Inadequate feeding practices is a key factor associated with under-nutrition in children younger than 5 years of age [104, 105]. Bidira et al. [106] educated caregivers of Ethiopian preschool children on healthy diet, nutrition, hygiene for 9 months. They showed that wasting and underweight significantly improved in the intervention group compared with controls. These findings highlight that the strategy of community-based nutritional education is effective in ameliorating nutritional status of young children [107].

### Infectious diseases. 1- Staphylococcus aureus; 2- Epstein-Barr virus

#### 1- Staphylococcus aureus

Methicillin-resistant Staphylococcus aureus (MRSA) can cause severe and highly prevalent diseases in the pediatric population. During the COVID-19 pandemic an increased rate of MRSA infections has been described in adults. In a study conducted in the period 2017–2020, which included the pandemic period, a similar epidemiologic trend in patients aged < 18 years, has not been confirmed [108]. Moreover, the efficacy against MRSA of several antibiotics was tested and linezolid and vancomycin were identified as the only antibiotics to which MRSA did not develop resistance. Of particular interest to the reader are the in-depth analysis of the factors related to methicillin resistance, as well as the antimicrobial susceptibility pattern according to methicillin resistance [109]. The authors conclude that surveillance of antimicrobial resistance is essential to improve infection control, antibiotic prescriptions, and preventions policies [110, 111].

#### 2- Epstein-Barr virus

Accomando et al. [112] report a child with symptomatic pancreatitis associated to Epstein-Barr virus infection. In this case, characterized by an enlarged pancreas visualized by abdominal ultrasound, the etiological diagnosis was only achieved by a positive serology for the presence of Epstein-Barr virus VCA IgM and IgG. The review of the few cases described in the literature draws attention to the importance of differential diagnosis that include specific serological research even in the absence of the classic clinical and haematological characteristics of Epstein-Barr virus infection [113]. Generally, Epstein-Barr virus-associated acute pancreatitis is characterized by a favorable prognosis, with a spontaneous resolution [114, 115].

### Metabolism. 1- Familial hypercholesterolemia; 2- Pompe disease

#### 1- Familial hypercholesterolemia

Familial hypercholesterolemia is the most common hereditary disorder of lipid metabolism causing life-long accumulation of low-density lipoprotein cholesterol [116]. In this article, Banderali et al. [117] highlight the main diagnostic strategies to identify this condition that, if left untreated, can lead to atherosclerosis and possible premature coronary heart disease and other vascular. Early detection of the condition is important to prevent complications and improve life expectancy. Cardiovascular events are rare among children and adolescents. Thus, identification of suggestive symptoms and family history is crucial. Some of the most typical signs of FH are: corneal arcus, xanthelasma and xanthomas [118]. Currently, screening tools are not available for pediatric patients, especially younger than 2 years old. Performing a cascade screening of close relatives, starting by drawing a pedigree and then identifying the subjects with a cardiovascular event or hypercholesterolemia (clinical or genetically defined) would be suitable. If familial hypercholesterolemia is reported, hypercholesterolemia or a cardiovascular event is found in all generations. The gold standard diagnostic tool of familial hypercholesterolemia includes the detection of familial hypercholesterolemia -causing mutation [119]. Further multidisciplinary team works are needed to identify optimal models of diagnosis and treatment of this life-threatening condition.

#### 2- Pompe disease

A review [120], enriched with the shared patient experience of the Italian Pediatric study group on immune response to enzymatic replacement therapy (ERT) with alglucosidase alfa in patients with Pompe disease, deals in depth with key elements of the complex and individualized management of immune response to ERT [121, 122]. Main objectives of the analysis, supported by useful diagrams, are identification of two types of immune reactions against ERT, quantification of risk factors for immunogenicity, choose of prophylaxis protocols in relation to cross reactive immunological material status, importance of immune-tolerance induction protocols in patients who develop antibodies during ERT, recommendations on the test to be performed before starting immunomodulation and tips on managing the frequent (up to 50%) hypersensitivity reactions during ERT. The authors conclude that further studies are needed to improve actual management protocols [123].

### Neonatology. 1- Preterm infant; 2- HIV infection; 3- Loeys-Dietz syndrome; 4- Hyperbilirubinemia; 5- Frenotomy; 6- Hypothermia

#### 1- Preterm infant

Parents of premature newborns admitted to neonatal intensive care unit (NICU) experience high levels of stress, determined both by the basic individual psychological well-being and the risk profile, the clinical complications, and the duration of the stay of their child. These conditions can have psychological consequences such as post-traumatic stress disorder [124, 125]. Salomè et al. [126] examined how the state of parental psychological well-being and stress experienced affect the appearance of post-traumatic stress disorder in the year following discharge. The authors use an up-to-date and valuable set of psychological tests, described in the article and useful to the reader for planning similar studies. The results identify a higher risk of post-traumatic stress disorder in mothers compared to fathers (55% vs. 20%), with specific correlations between specific experiences and emotional reactions. In addition, they provide interesting research cues and identification of targeted intervention to reduce the risk of a post-traumatic stress disorder in parents of the preterm infants [127]. In the position paper of the Italian scientific societies of pediatric area [128], recommendations are suggested regarding the important step of the introduction of complementary feeding in preterm infants. The main objectives are to reduce the risk of extrauterine growth restriction and the features of altered body composition with reduction of free mass and increased adiposity [129–131]. Main recommendations include to start complementary feeding between 5 and 8 months of chronological age, consider the limit of 3 months corrected age to ensure the acquisition of developmental skills allowing the consumption of solid foods, consider multidisciplinary assessment before starting complementary feeding in case of oral dysfunction or comorbidities, type (included allergenic food), sequence and speed of introduction of food, vitamins, and micronutrients. Exclusive breastfeeding, mixed feeding or standard infant formula enriched with long chain polyunsaturated fatty acids should be preferred for infants without extrauterine growth restriction while extrauterine growth restriction infants or who is at risk of long-term growth failure may be fed with fortified human milk or formula adapted for preterm infants as long as to gain an optimal weight for corrected age.

#### 2- HIV infection

In West Amhara, Ethiopia an unmatched case–control study from 2016 to 2020, aimed to identify determinants of HIV infection among children born to HIV positive mothers on the prevention of the mother -child transmission program at referral hospitals [132]. The region is characterized by an important HIV public health problem with a high transmission rate. The identified determinants were home delivery, mixed feeding, poor maternal antiretroviral drug adherence, advanced World Health Organization (WHO) clinical stage, poor nevirapine adherence and late enrollment of the infant. The authors conclude that Minister of Health and non-governmental organizations should work on mobilization of the community and awareness creation on the important of exclusive breast feeding, drugs adherence, on benefits of health institutional delivery as well as the risk of homedelivery. Public health interventions are very important tools to reduce mother-child transmission of HIV [133–135].

#### 3- Loeys-Dietz syndrome

Loeys-Dietz syndrome is a rare connective tissue disorder related to a pathogenic variant in TGFBR1, TGFBR2, SMAD2, SMAD3, TGFB2 or TGFB3 genes. It involves the aorta with progressive dilatation, craniofacial skeleton, joints, skin, and is associated with hypotonia and motor delay [136, 137]. In a first patient [138] a trio based Whole Exome Sequencing found a novel, heterozygote, missense, de novo variant in the TGFBR2 gene. In a second patient [138], with a suspicious family history, a genetic panel for connective tissue disorders identified a pathogenetic variant in TGFB3 gene. Only in the first male patient the aortic aneurysm progressed despite of a low-dose angiotensin receptor blocker therapy, while the second female patient showed no aortic abnormalities. An early diagnosis of Loeys-Dietz syndrome implies a potential modification of the natural history of the disease with early interventions on its complications [139].

#### 4- Hyperbilirubinemia

A meta-analysis evaluates, in case of neonatal indirect hyperbilirubinemia in treatment with phototherapy, the use of ursodeoxycholic acid as adjunctive therapy [140]. Ursodeoxycholic acid is classically used for the management of cholestatic liver pathology through increased production of bile, the removal of the most toxic components of bile acids and additional hepatoprotective and neuroprotective effects [141–143]. The five selected studies from scientific literature suggest that the addition of ursodeoxycholic acid to phototherapy could reduce phototherapy duration by almost 18 h compared to phototherapy only. It also resulted in a lower mean of total serum bilirubin in the 48 h post-treatment, especially in Asian countries. Two infants with isolated severe unconiugated hyperbilirubinemia related to Gilbert syndrome have been reported [144]. In both infants the prolonged indirect hyperbilirubinemia associated with the finding of a mutation p.Pro364Leu in the bilirubin uridine diphosphate-glucuronosyltransferase gene raised the initial suspicion of Crigler-Najjar syndrome type II. Both infants were treated with phototherapy and phenobarbital, achieving a normalization of bilirubin levels at 1 and 5 months respectively [145–147]. The authors suggest that in these patients the concomitant breastfeeding jaundice was not a possible explanation of the clinical picture since a brief interruption of breastfeeding was not effective.

#### 5- Frenotomy

In a prospective observational cohort study, Dall’Olio et al. [148] evaluated diode laser frenotomy [149] in fifty-five newborns with ankyloglossia with or without difficult breastfeeding. Important functional consequences of ankyloglossia were manifested by 20 to 50% of cases adversely affecting the nutrition and growth of the newborn and the degree of adhesion of the mother to breastfeeding, her psychological well-being and bonding [150, 151]. The suggestions of the clinical tools used for evaluation of ankyloglossia, quality of breastfeeding, and perioperative pain of the newborn are useful. Short duration of the diode laser frenotomy, minimal reported complications, short hospital stay, and positive effects on breastfeeding, are elements in favor of the implementation of this procedure.

#### 6- Hypothermia

A single center, parallel-group, and no-blinded randomized controlled [152], has been conducted in a level III, and academic neonatal intensive care unit in China from 2020 to 2021, to evaluate the effect of early vs. delayed enteral nutrition on the incidence of feeding intolerance and secondary outcome including the incidences of late-onset bloodstream infection, hypoglycemia, survival at neonatal discharge, duration of parenteral nutrition, duration until attainment of full enteral feeds, length of hospital stay and body weight gain, in newborns treated with therapeutic hypothermia. Feeding strategy consisted of minimal enteral feeding and slow speed of increasing milk feeds. Early enteral nutrition was performed during therapeutic hypothermia /rewarming period while delayed enteral nutrition was performed after the therapeutic hypothermia phase. This study showed that the average time of parenteral nutrition, reaching full enteral feeds and hospital stay were shorter in the early enteral nutrition group compared with the delayed group with significant differences [153–155].

### Neurology. 1- Drooling; 2- Alternating hemiplegia; 3- Autism; 4- Pontocerebellar hypoplasia; 5- Vertigo; 6- Idiopathic intracranial hypertension; 7- Pressure palsies; 8- Epilepsy; 9- Diencephalic syndrome

#### 1- Drooling

Drooling is defined as the involuntary loss of saliva and oral content. While most children reach the salivary continence by age 15–18 months, the persistence of drooling is common in children with neurological disorders, like cerebral palsy (CP) and is associated with oral motor dysfunction, dysphagia, and/or intraoral sensitivity disorder [156]. Several therapeutic interventions are used to reduce or eliminate drooling. These include surgery, drugs, botulinum toxin, physical therapies to improve sensory function, behavioral interventions, appliances placed in the oral cavity, and acupuncture [157]. There is no consensus on which interventions are safe and effective in managing drooling in children with CP. As the salivary glands are controlled by the parasympathetic autonomic nervous system, the anticholinergic drugs are indeed widely used to reduce the volume of saliva. Glycopyrrolate is the only oral formulation including an anticholinergic agent approved by the United States Food and Drug Administration to treat drooling in children 3–16 years-old which inhibits the acetylcholine receptors on peripheral tissues and reduces the saliva rate production [158]. Lovardi et al. [159] described a case series of eighteen children (median age 17 months, range 2–36 months) with CP or genetic/malformative syndrome, who have been administered oral glycopyrrolate (0.065 mg/kg/die in 3 daily doses) for drooling control. The Drooling Impact Scale was administered at time O and after 1 month. Results showed a significant reduction of the Drooling Impact Scale after 1 month (89 versus 61; p < 0.001), with few adverse effects and an overall response to treatment equal to 94%. Further studies are needed to confirm these results.

#### 2- Alternating hemiplegia

Alternating hemiplegia of childhood is an uncommon complex disorder, which was first described in late 1960s by Verret and Steele [160]. Alternating hemiplegia of childhood is characterized by paroxysmal episodes of repeated, transient paresis involving either or both sides of the body and usually presents before age 18 months. The diagnosis of alternating hemiplegia of childhood is mainly clinical but may be supported by molecular analysis. Although the pathophysiologic mechanism of the disorder is partially unclear, it is well known that a relevant role is played by mutations in ATP1A2 and ATP1A3 genes, which encode two different alpha subunits of the Na+/K + ATPase transmembrane ion pump, respectively [161]. Pavone et al. [162] reported on the clinical and genetic findings of a couple of twins and a couple of siblings with alternating hemiplegia of childhood from two different Italian families affected. In the twins a pathogenic variant in ATP1A3 gene (c.2318 A > G) was detected. In the siblings, the younger brother showed a novel GRIN2A variant (c.3175 T > A), while the older one carried the same GRIN2A variant, associated with two likely pathogenetic variants in SCNIB (c.632 > A) and KCNQ2 (1870 G > A) genes, which have been implicated in childhood epilepsies. This report provides additional information about alternating hemiplegia of childhood, showing that the variability of clinical features is mirrored by an unexpected genetic heterogeneity. Clinical signs of alternating hemiplegia of childhood usually follow a sequential pattern, in which the paroxysmal episodes are triggered by precipitating factors such as environmental stress, bathing or other events. Non-paroxysmal features include developmental delay/intellectual disability, epilepsy, autonomic dysfunctions, abnormal eye involvement, movement disorders, ataxia, dystonia, and choreoathetosis [163].

#### 3- Autism

Autism spectrum disorder is a term used to describe a set of social communication deficits and repetitive sensory–motor behaviors. It is a neurological developmental disorder, and is characterized by a complex etiology involving genetic, environmental, and biochemical factors [164]. The autism spectrum includes several disorders, such as the Autistic disorder, the Rett disorder, the Asperger syndrome, and the pervasive developmental disorder. Although individuals with autism spectrum disorder are very different from one another, the disorder affects social interactions, verbal and nonverbal social communication skills, as well as intelligence and motor functions, usually originating unusual interests and repetitive behaviors [165]. Epidemiological surveys have shown a rapid increase in autism spectrum disorder prevalence rate. Besides the real increase in prevalence, a variety of other reasons may contribute to the disorder, such as a broader definition of autism spectrum disorder, changes in diagnostic criteria and screening tools, and finally an increased awareness of autism spectrum disorder [166]. In this scenario, Salari et al. [167] conducted a systematic review and meta-analysis from 2008 to July 2021 finalized to clarify the global prevalence of autism spectrum disorder [159] The results show that the world prevalence of autism spectrum disorder is 0.6% (95% confidence interval: 0.4;1%), with slight differences between Asia (0.4%), America (1%), Europe (0.5%), Africa (1%) or Australia (1.7%). Standardized screening for autism spectrum disorder with ongoing developmental surveillance continues to be recommended in primary care at 18 and 24 months of age, because autism spectrum disorder is common, can be diagnosed as young as 18 months of age, and medical interventions may significantly affect neurological compromise [168]. These data should be disseminated by health policymakers in order to implement appropriate planning and interventions.

#### 4- Pontocerebellar hypoplasia

Pontocerebellar hypoplasia includes a heterogeneous group of neurodegenerative disorders with prenatal onset, characterized by severe microcephaly, global developmental delay and radiological manifestation, such as hypoplasia of pons and cerebellum [169]. At least 21 pontocerebellar hypoplasia -related genes are currently listed in the OMIM-database and 15 types of pontocerebellar hypoplasia are known, all characterized by patients’ motor and cognitive impairment. However, the clinical presentation can be very different, ranging from lethal neonatal subtypes to milder variants with survival up to adolescence [170]. The study from Bilge et al. consisted of the description of different clinical and radiological manifestations of six genetically diagnosed patients with pontocerebellar hypoplasia, highlighting the differences in onset and progression of the symptoms [171]. The genetic mutations found in 4 out 6 cases were well known variants that had been previously reported in the literature, while an additional subject was homozygous for the TBC1D23 gene mutation (c.1263 + 1G > A), which was a novel variant never reported until now. Although the last case had the same clinical features, the whole-exome sequencing revealed compound heterozygous mutations in the BRF1 gene, which is also associated with cerebellofaciodental syndrome. Common problems in pontocerebellar hypoplasia include sleep apnea, feeding problems, epilepsy, movement disorders, rhabdomyolysis, and extremely elevated serum creatinine kinase, especially during infections [170]. There is no definite treatment for any type of pontocerebellar hypoplasia, and management is supportive in all types and subtypes.

#### 5- Vertigo

Vertigo is a disorder of space sensitivity characterized by an illusory and unpleasant sensation of body movement toward the environment or vice versa. Vertigo is characterized by different clinical features, therefore it is often difficult for the single physician to identify its etiopathogenesis, especially when the vertigo is the only clinical presenting sign [172]. Compared to adults, vertigo is not so common in children and adolescents. Indeed, while adults surveys reported a one-year prevalence of 23% for unspecified dizziness and 5% for vestibular vertigo, in pediatric patients a prevalence of only 0.4% for nonspecific dizziness, 0.03% for peripheral, and 0.02% for central vestibulopathy is reported [173]. Nevertheless, it is a common reason for emergency department presentation, both alone or associated with other symptoms. Pellegrino et al. [174] reported on the etiopathology of neurological vertigo in childhood and adulthood, highlighting the characteristics which may lead clinicians to a proper diagnosis, and proposed a diagnostic algorithm to support the approach to patients with isolated vertigo, both in pediatric and adult age. The most common cause of isolated vertigo in the pediatric population is vestibular migraine, followed by benign paroxysmal vertigo in childhood. Other causes of vertigo are orthostatic hypotension, vestibular neuritis and vestibular paroxysmia, neurovascular diseases, tumors and demyelinating [175]. Authors underline that the outcome of most neurological vertigos in childhood is good, in contrary to what is reported in adults.

#### 6- Idiopathic intracranial hypertension

Idiopathic intracranial hypertension is very rare in the pediatric age, and etiology is still largely unknown. Del Monte et al. [176] presented a case report of an 8-month old male affected by idiopathic intracranial hypertension, and provide discussion on what literature suggests about optimal management also including the therapeutic strategies in idiopathic intracranial hypertension. The presented case was admitted with vomit, anorexia, irritability and bulging anterior fontanel. Brain magnetic resonance and cerebrospinal fluid analysis were negative. After diagnosis and treatment with acetazolamide and corticosteroids, progressive resolution of symptoms was observed. Pseudotumor cerebri syndrome is a rare condition characterized by elevated intracranial pressure, normal cerebrospinal fluid analysis in the absence of intracranial lesions on neuroimaging. Most common type of pseudotumor cerebri syndrome in both children and adults is the primary type known as idiopathic intracranial hypertension [177]. Secondary types can result from vascular malformations, underlying systemic conditions or drugs. Female gender, obesity, and polycystic ovary represent risk factors for idiopathic intracranial hypertension in adolescents, but not in younger ones [178]. Clinical manifestations of idiopathic intracranial hypertension vary with age. In pediatric populations, symptoms are often unspecific, including vomiting, headache, irritability, hyporeactivity, anorexia, sleep disruption, head tilting, papilledema and bulging fontanelle in infants. Based on adults’ treatment guidelines, also in children the drug of choice is acetazolamide, that could be associated with intravenous corticosteroids if severe visual impairment is present as well. So far, the moment, acetazolamide alone is preferred because of fewer side effects. Most common complications of idiopathic intracranial hypertension are vision loss and relapse, especially in the first 18 months after diagnosis [179]. Early diagnosis, treatment and strict follow-up of complications may help in reducing the risk of relapse and may prevent vision loss.

#### 7- Pressure palsies

Karlinsky et al. [180] reported their experience about an unusual case of hereditary neuropathy with liability to pressure palsies (HNPP) who had limping as a major presenting symptom. HNPP is an autosomal dominant disease characterized by recurrent, episodic demyelinating neuropathy caused by 17p11.2 chromosomal deletion encompassing the PMP22 gene. The onset is acute, usually in adulthood, and involves a single nerve, most frequently peroneal and ulnar, with a non-painful focal sensory and motor neuropathy [181]. The onset in children most commonly involves peroneal nerve palsy and brachial plexus palsy [182]. Other signs are previous atrophy, focal weakness or sensory loss, reduction of tendon reflexes and mild to moderate *pes cavus* foot deformity [183]. The definitive diagnosis is provided by genetic analysis, and treatment is mostly supportive. Prognosis is good with an expected full recovery within months or days in best case scenarios. In rare cases a persistence of severe symptoms is reported. Since presenting phenotypes can be extremely variable, the authors describe their experience that led to the identification of 3 additional cases within the pedigree, providing family members with an accurate genetic counseling.

#### 8- Epilepsy

Febrile infection-related Epilepsy Syndrome (FIRES) is an uncommon but severe disorder manifesting a prior febrile infection starting between 2 weeks and 24 h before the onset of the refractory status epilepticus with or without fever at the onset of status epilepticus. Symptoms of FIRES had been previously named as “acute encephalitis with refractory, repetitive partial seizures” or as “fever-induced refractory epileptic encephalopathy in school age children”. In this literature review, Pavone et al. [184] presented two cases with FIRES and discussed about this rare even though severe disorder. Both cases presented with the classical FIRES symptoms and reported moderate-severe cognitive impairment and persistence of seizures. Clinical manifestations of FIRES are focal seizures in the acute phase with possible secondary generalization, sometimes associated with pallor, apnea, and cyanosis [185]. Current guidelines for diagnosis of FIRES are not yet available [186]. Different treatment options have been reported, usually suggested during the second phase of the disease. Most used drugs are intravenous benzodiazepines as midazolam, clonazepam, lorazepam and diazepam in association with standard anticonvulsant drugs, as oral levetiracetam, valproic acid, and lacosamide [186]. Alternative therapeutic strategies are ketogenic diet immunomodulatory and intravenous steroids at high-dose or immunoglobulins, plasmapheresis and other agents [187]. Clinical outcomes usually see the persistence of seizure episodes, intellectual disability or, in more severe cases, vegetative state [188]. FIRES is an uncommon and not yet totally understood entity. Since many aspects of this disease such as pathogenesis and treatment still need to be clarified, further future studies are needed. Epilepsy is a common and serious multifactorial neurologic disease with a strong genetic component [189]. Ghazala et al. [190] investigated the SCN1A-A3184G polymorphism among Egyptian children and adolescents with non-lesional epilepsy conducting a prospective case-control observational study. Neuronal voltage-gated sodium channels are involved in the generation and propagation of the action potentials within the neurons, acting on membrane permeability to sodium ions that allows ions diffusion down an electrochemical gradient till the sodium equilibrium potential [191]. There is evidence about the role of the neuronal voltage-gated sodium channels polymorphisms in the epilepsy pathogenesis that causes a spectrum of epilepsy syndromes. For this reason, sodium-channel blockers such as carbamazepine, oxcarbazepine, phenytoin, lamotrigine, lacosamide and lidocaine are some of the most common therapeutic options for epilepsies due to genetic channelopathies. The SCN1A-A3184G (p.Thr1067Ala) polymorphism has been suggested to be linked with the epilepsy risk in several non-Caucasian populations [192]. In their study, the authors extracted and analyzed genomic DNA controls and cases. Results of the study report insignificant differences between epilepsy cases and the control group regarding the frequency of SCN1A-A3184G genotypes and allele. Authors also suggest that the identification of SCN1A-A3184G genotypes might help to choose the most suitable antiepileptic drugs. Further studies on a larger scale are needed to better clarify the matter.

#### 9- Diencephalic syndrome

Trapani et al. [193] provide relevant insights on the diencephalic syndrome, a rare childhood disease causing failure to thrive. They presented three children who were admitted for progressive weight loss. Diencephalic syndrome is provoked by a hypothalamic dysfunction caused by a tumor that may involve thalamus/hypothalamus and optic chiasm [194]. Presenting symptoms of diencephalic syndrome may be unspecific. Most common features are severe emaciation with normal caloric intake, locomotor hyperactivity and euphoria, pallor without anemia, hypoglycemia and hypotension [195]. Neurological symptoms typically appear later and include nystagmus, strabismus and visual loss; intracranial hypertension causing recurrent vomiting can be also present, without signs of psychomotor impairment. Diagnosis can be tricky due to the non-specific signs of presentation. Gold standard for radiological detection of brain mass is magnetic resonance. Since surgical removal is not always feasible, chemotherapy based on vincristine, carboplatin and/or etoposide is one of the main therapeutic options [196]. The first case is a 26-month-old boy with persistent failure to gain weight despite adequate caloric intake, no gastrointestinal or other symptoms and negative blood exams about possible malabsorption or vitamin deficiencies. Hormone evaluations and other organ functions were also normal. Brain magnetic resonance was finally performed and detected a large low-grade astrocytoma located in the supra-sellar region. The second case is a 14-month-old girl admitted for poor weight gain, with normal caloric intake. Same investigations were conducted, as well as a brain magnetic resonance showing supra-sellar mass involving hypothalamic-pituitary region, later identified as a low-grade astrocytoma. Finally, the third case is a 16-month-old male who was admitted for severe failure to thrive. After initial diagnostic workup, in the suspicion of diencephalic syndrome, brain imaging identified a large multilobate pseudo-cystic suprasellar lesion in the hypothalamic-pituitary and chiasmatic region. Biopsy of the lesion led to histological diagnosis of pilomyxoid astrocytoma. In this interesting series authors clarified useful elements for the identification of this uncommon syndrome in which subtle presenting symptoms can often lead to a delayed diagnosis.

### **Oncology. 1- Care burden**

#### 1- Care burden

X 400.000 children and adolescents between ages of 0 and 19 each year. The most common types of childhood cancers include leukemias, brain cancers, lymphomas and solid tumors, such as neuroblastoma and Wilms tumor [197]. Scientific advancement has led to an increase in the survival rate of children with cancer and, as a result, living with cancer affects different aspects of life of these children and their caregivers [198]. Chaghazardi et al. [199] determined the level of care burden and the factors associated with it among the caregivers of children with cancer, and have highlighted that the majority of caregivers experience moderate to high care burden. Following the diagnosis, caregivers have to face several challenges such as ignorance, instability, anxiety, helplessness, confusion, and stress. High care burden can affect the quality of care provided to the patient and thus exacerbate their condition, leading to an increase of care burden in a vicious circle [200]. For this reason, identifying and helping caregivers with care burden play a crucial role in improving the quality of care to affected children. Authorities need to take family-centered measures to reduce the caregiver burden, referring low-income caregivers to support organizations, providing counseling services, and holding training workshops on caring for children with cancer.

### Pneumology. 1- E-cigarettes; 2- Cystic fibrosis

#### 1- E-cigarettes

Electronic cigarettes are devices that heat liquid substances that may contain nicotine that can substitute burned cigarettes. Virgili et al. [201] presented the effects of e-cigarette use on health and pointed out the potential damages arising also from second- and third-hand exposure to smoke. Even though e-cigarettes are considered to be less harmful than burned cigarettes, the Center for Disease Control and Prevention defines them as unsafe and potentially dangerous for brain development and increasement of risk for addiction [202]. Damages of vaping on the respiratory system are analogous to the ones of smoking: chronic inflammation of bronchial mucosa and lung epithelium injury are typically observed. Due to the increasing popularity of vaping products, a new type of lung damage has been described: E-cigarette or Vaping Associated Lung Injury (EVALI). The diagnosis is made after the exclusion of all other potential causes of lung injury when pulmonary infiltrates are found at chest imaging in patients using e-cigarettes and related products 90 days prior to symptom onset [203]. Symptoms of presentation are usually cough, dyspnea and tachycardia and chest imaging usually shows ground glass appearance or dense consolidations. Therapy is based on antibiotics and steroids and, when needed, high-flow oxygen therapy, noninvasive ventilation or mechanical ventilation [204]. Effects on the cardiovascular system include myocardial infarction, hypertension and tachycardia. Neurological effects are the ones observed in nicotine users and include craving and withdrawal symptoms. Gastroenteric effects are an increased risk for gastro-esophageal reflux and for esophagitis exacerbations. Overall, due to its various harmful effects, vaping could be useful only if used as a smoking-cessation device. It is important for institutions to keep restrictions and prevention strategies and for educators and pediatricians to increase awareness in children and their families. During the last years the use of E-cigarettes has increased dramatically in Italy [205]. Casamento Tumeo et al. [206] described a case of E-cigarette or EVALI in a 15-year-old girl. The girl presented to the emergency room with severe dyspnea and a SatO2 of 75% and chest auscultation revealed bilateral wheezes with prolonged expiration. After admission, oxygen supplementation was started and a chest tomography was performed, showing central ground glass pattern with peripheral sparing. She was started on antibiotic therapy with clarithromycin and ceftriaxone. High Flow Nasal Cannula, systemic steroid and nebulized salbutamol, ipratropium and corticosteroids. The girl had a history of asthma and multiple allergies and she was a tobacco and E-cigarette smoker. Dyspnea started 4 days before the admission to the emergency department, but no fever or cough were present. Prior to admission, antibiotic therapy was prescribed with no improvements. After admission and initial treatments, further examinations were performed. Among them, pulmonary function tests showed a restrictive pattern, without bronchodilator reversibility. Diagnostic criteria for identification of EVALI include the use of e-cigarettes 90 days prior to the onset of symptoms accompanied by specific radiological findings (typically ground glass opacities at chest tomography) [207]. EVALI is more common in patients with underlying asthma [208]. Even if its cause is still not yet known, in recent years, Vitamin E has been reported in the broncho-alveolar lavage of patients affected by EVALI, thus identifying it as possible pathogen [209]. Since its relatively recent spread, the use of E-cigarette still needs to be studied in order to better understand the impact and potential damages on health.

#### 2- Cystic fibrosis

Cystic fibrosis (CF) is a life-limiting autosomal recessive disorder due to variants in the Cystic Fibrosis Transmembrane Conductance Regulator (CFTR) gene. CFTR protein is responsible for chloride ion transport across apical epithelial cells in tissues of the airway, intestine, pancreas, kidney, sweat gland, and male reproductive tract [210]. Nowadays, CFTR modulator therapies, targeting the basic CF molecular defect, have been developed for specific CFTR variants and are associated with an improvement in health outcomes, including respiratory function, nutritional status and enhanced quality of life. The quality of life and the survival of patients with CF significantly improved since these drugs have been available and several progresses are being made in the development of such drugs [211]. Nevertheless, lung disease remains the most common cause of death in CF patients and symptomatic mucolytic drugs are crucial for reducing secretion build-up, preventing infections and slowing lung damage. Terlizzi et al. [212] have summarized the current knowledge on dornase alfa (DNAse) in the treatment of CF lung disease, showing the positive effects of this drug on lung ventilation homogeneity. To date DNAse is the only mucus degrading agent that has proven efficacy in CF [213], by reducing the number of pulmonary exacerbations and improving Forced Expiratory Volume in the 1st second and lung clearance index in patients with CF, in the absence of significant side effects. That is why an early use would be desirable in CF children from 6 years of age, especially in the presence of an abnormal lung clearance index.

## Conclusions

Over the past year, advances in epidemiology, pathophysiologic mechanisms and prevention, have deepened our understanding of pediatric diseases. We think that developments achieved in 2022 can guide to improve and expand the horizon of care for children.

## Data Availability

Data sharing is not applicable to this article as no datasets were generated or analysed during the current study.

## Abbreviations

BMI: Body Mass Index
CP: Cerebral palsy
COVID-19: Coronavirus Disease
CF: Cystic Fibrosis
CFTR: Cystic Fibrosis Transmembrane Conductance Regulator
DNAse: Dornase alfa
ERT: Enzymatic replacement therapy
FIRES: Febrile infection-related epilepsy syndrome
HNPP: Hereditary neuropathy with liability to pressure palsies
MRSA: Methicillin-resistant Staphylococcus aureus
NICU: Neonatal intensive care unit
ST: screening tool
SCE: self-core evaluation
SARS-CoV-2: Severe Acute Respiratory Syndrome Coronavirus-2
USP7: Ubiquitin-Specific Peptidase 7 gene
EVALI: Vaping Associated Lung Injury
WHO: World Health Organization

## References

[1] Ricci G, Andreozzi L, Cipriani F, Giannetti A, Gallucci M, Caffarelli C (2019). Wheat allergy in children: a comprehensive update. Med (Kaunas).

[2] Mastrorilli C, Cardinale F, Giannetti A, Caffarelli C (2016). Pollen-food allergy syndrome: a not so rare disease in childhood. Med (Kaunas).

[3] Berni Canani R, Caffarelli C, Calvani M, Martelli A, Carucci L, Cozzolino T (2022). Diagnostic therapeutic care pathway for pediatric food allergies and intolerances in Italy: a joint position paper by the italian society for Pediatric Gastroenterology Hepatology and Nutrition (SIGENP) and the italian society for Pediatric Allergy and Immunology (SIAIP). Ital J Pediatr.

[4] Caffarelli C, Ricò S, Varini M, Povesi-Dascola C, Mastrorilli C (2016). Skin prick test and development of tolerance in egg allergy. Pediatr Allergy Immunol.

[5] Caffarelli C, Ricò S, Rinaldi L, Povesi Dascola C, Terzi C, Bernasconi S (2012). Blood pressure monitoring in children undergoing food challenge: association with anaphylaxis. Ann Allergy Asthma Immunol.

[6] Caffarelli C, Dondi A, Povesi Dascola C, Ricci G (2013). Skin prick test to foods in childhood atopic eczema: pros and cons. Ital J Pediatr.

[7] Galli E, Fortina AB, Ricci G, Maiello N, Neri I, Baldo E (2022). Narrative review on the management of moderate-severe atopic dermatitis in pediatric age of the italian society of Pediatric Allergology and Immunology (SIAIP), of the italian society of Pediatric Dermatology (SIDerP) and of the italian society of pediatrics (SIP). Ital J Pediatr.

[8] Di Rienzo V, Cadario G, Grieco T, Galluccio AG, Caffarelli C, Liotta G (2014). Sublingual immunotherapy in mite-sensitized children with atopic dermatitis: a randomized, open, parallel-group study. Ann Allergy Asthma Immunol.

[9] Caffarelli C, Dascola CP, Peroni D, Ricò S, Stringari G, Varini M (2014). Airway acidification in childhood asthma exacerbations. Allergy Asthma Proc.

[10] Caffarelli C, Calcinai E, Rinaldi L, Povesi Dascola C, Terracciano L, Corradi M (2012). Hydrogen peroxide in exhaled breath condensate in asthmatic children during acute exacerbation and after treatment. Respiration.

[11] Zinelli C, Caffarelli C, Strid J, Jaffe A, Atherton DJ (2009). Measurement of nitric oxide and 8-isoprostane in exhaled breath of children with atopic eczema. Clin Exp Dermatol.

[12] Riscassi S, Corradi M, Andreoli R, Maccari C, Mercolini F, Pescollderungg L (2022). Nitric oxide products and aldehydes in exhaled breath condensate in children with asthma. Clin Exp Allergy.

[13] Manna A, Caffarelli C, Varini M, Povesi Dascola C, Montella S, Maglione M (2012). Clinical application of exhaled nitric oxide measurement in pediatric lung diseases. Ital J Pediatr.

[14] McCrossan P, Mallon O, Shields MD, Russell C, Kennedy L, O’Donoghue D (2022). How we teach children with asthma to use their inhaler: a scoping review. Ital J Pediatr.

[15] Mastrorilli C, Pecoraro L, Arasi S, Barni S, Caminiti L, Castagnoli R (2022). Rare allergic Diseases Commission of the italian society of Pediatric Allergy and Immunology. Pediatric hypersensitivity pneumonitis: literature update and proposal of a diagnostic algorithm. Ital J Pediatr.

[16] Wawszczak M, Bielecka T, Szczukocki M (2021). Hypersensitivity pneumonitis in children. Ann Agric Environ Med.

[17] Bush A, Cunningham S, de Blic J, Barbato A, Clement A, Epaud R (2015). European protocols for the diagnosis and initial treatment of interstitial lung disease in children. Thorax.

[18] Toyotome T, Satoh M, Yahiro M, Watanabe A, Nomura F, Kamei K (2014). Glucoamylase is a major allergen of Schizophyllum commune. Clin Exp Allergy.

[19] Cossovel F, Trombetta A, Ramondo A, Riccio G, Ronfani L, Saccari A (2022). Intranasal dexmedetomidine and intranasal ketamine association allows shorter induction time for pediatric sedation compared to intranasal dexmedetomidine and oral midazolam. Ital J Pediatr.

[20] Li BL, Luo H, Huang JX, Zhang HH, Paquin JR, Yuen VM (2022). Using intranasal dexmedetomidine with buccal midazolam for magnetic resonance imaging sedation in children: a single-arm prospective interventional study. Front Pediatr.

[21] Gu H, Miao L, Bai J, Lu G, Lei Q, Yang L (2022). Combined use of intranasal dexmedetomidine and an oral novel formulation of Midazolam for sedation of young children during brain MRI examination: a prospective, single-center, randomized controlled trial. BMC Anesthesiol.

[22] Cui Y, Gong T, Mu Q, Wu Q, Kang L, Chen Q et al. Predictors of pediatric sedation failure with initial dose of intranasal dexmedetomidine and oral midazolam. Pediatr Res 2023 Jul 28. 10.1038/s41390-023-02758-0. Online ahead of print. 10.1038/s41390-023-02758-0 37507474

[23] Candelino M, Tagi VM, Chiarelli F (2022). Cardiovascular risk in children: a burden for future generations. Ital J Pediatr.

[24] Schipper HS, de Ferranti S (2022). Atherosclerotic cardiovascular risk as an emerging priority in pediatrics. Pediatrics.

[25] Jacobs DR, Woo JG, Sinaiko AR, Daniels SR, Ikonen J, Juonala M (2022). Childhood cardiovascular risk factors and adult cardiovascular events. N Engl J Med.

[26] Pool LR, Aguayo L, Brzezinski M, Perak AM, Davis MM, Greenland P (2021). Childhood risk factors and adulthood cardiovascular disease: a systematic review. J Pediatr.

[27] Pensabene M, Di Pace MR, Baldanza F, Grasso F, Patti M, Sergio M (2022). Quality of life improving after propranolol treatment in patients with infantile hemangiomas. Ital J Pediatr.

[28] Pahl KS, McLean TW (2022). Infantile hemangioma: a current review. J Pediatr Hematol Oncol.

[29] Lorusso B, Cerasoli G, Falco A, Frati C, Graiani G, Madeddu D (2022). Β-blockers activate autophagy on infantile hemangioma-derived endothelial cells in vitro. Vascul Pharmacol.

[30] Léauté-Labrèze C, Frieden I, Delarue A (2023). Early initiation of treatment with oral propranolol for infantile hemangioma improves success rate. Pediatr Dermatol.

[31] Retrosi C, Diociaiuti A, De Ranieri C, Corbeddu M, Carnevale C, Giancristoforo S (2022). Multidisciplinary care for patients with epidermolysis bullosa from birth to adolescence: experience of one italian reference center. Ital J Pediatr.

[32] Mauritz P, Jonkman MF, Visser SS, Finkenauer C, Duipmans JC, Hagedoorn M (2019). Impact of painful wound care in epidermolysis bullosa during childhood: an interview study with adult patients and parents. Acta Derm Venereol.

[33] Fine J-D, Johnson LB, Weiner M, Suchindran C (2008). Cause-specific risks of childhood death in inherited epidermolysis bullosa. J Pediatr.

[34] Montaudié H, Chiaverini C, Sbidian E, Charlesworth A, Lacour JP (2016). Inherited epidermolysis bullosa and squamous cell carcinoma: a systematic review of 117 cases. Orphanet J Rare Dis.

[35] Vahlquist A, Fischer J, Törmä H (2018). Inherited nonsyndromic ichthyoses: an update on pathophysiology, diagnosis and treatment. Am J Clin Dermatol.

[36] Serra G, Memo L, Cavicchioli P, Cutrone M, Giuffrè M, La Torre ML (2022). Novel mutations of the ABCA12, KRT1 and ST14 genes in three unrelated newborns showing congenital ichthyosis. Ital J Pediatr.

[37] Akiyama M (2010). ABCA12 mutations and autosomal recessive congenital ichthyosis: a review of genotype/phenotype correlations and of pathogeneticoncepts. Hum Mutat.

[38] Serra G, Memo L, Coscia A, Giuffré M, Iuculano A, Lanna M (2021). Recommendations for neonatologists and pediatricians working in first level birthing centers on the first communication of genetic disease and malformation syndrome diagnosis: consensus issued by 6 italian scientific societies and 4 parents’ associations. Ital J Pediatr.

[39] Nicolucci A, Maffeis C (2022). The adolescent with obesity: what perspectives for treatment?. Ital J Pediatr.

[40] Kelly AS (2023). Current and future pharmacotherapies for obesity in children and adolescents. Nat Rev Endocrinol.

[41] Wu Z, Gao Z, Qiao Y, Chen F, Guan B, Wu L (2023). Long-term results of bariatric surgery in adolescents with at least 5 years of follow-up: a systematic review and meta-analysis. Obes Surg.

[42] Martinelli V, Singh S, Politi P, Caccialanza R, Peri A, Pietrabissa A (2023). Ethics of bariatric surgery in adolescence and its implications for clinical practice. Int J Environ Res Public Health.

[43] Gaeta F, Conti V, Pepe A, Vajro P, Filippelli A, Mandato C (2022). Drug dosing in children with obesity: a narrative updated review. Ital J Pediatr.

[44] Bradley SJ, Nelson JD, Barnett E, Cantey JB, Kimberlin DW, Palumbo PE (2021). Nelson’s Pediatric Antimicrobial Therapy. Am Acad Pediatr.

[45] Garner MP, Onuoha CP, Fenn NE (2021). Low-molecular-weight heparin and Fondaparinux use in pediatric patients with obesity. Ann Pharmacother.

[46] Sadat-Ali M, AlTabash KW, Al-Turki HA, AlMousa SA, AlSayed HN (2021). Time out: should vitamin D dosing be based on patient’s body mass index (BMI): a prospective controlled study. J Nutr Sci.

[47] Ferrari V, Stefanucci S, Ciofi D, Stagi S (2022). Analysis of the timing of puberty in a recent cohort of italian girls: evidence for earlier onset compared to previous studies. J Pediatr Adolesc Gynecol.

[48] Li W, Liu Q, Deng X, Chen Y, Liu S, Story M (2017). Association between obesity and puberty timing: a systematic review and meta-analysis. Int J Environ Res Public Health.

[49] Wang Y, Dinse GE, Rogan WJ (2012). Birth weight early weight gain and pubertal maturation: a longitudinal study. Pediatr Obes.

[50] Ferrari V, Stefanucci S, Ferrari M, Ciofi D, Stagi S, on the behalf of the Tuscany Menarche Study Group (2022). Retrospective longitudinal analysis of the effects of postnatal weight gain on the timing and tempo of puberty and menarche in a cohort of italian girls. Ital J Pediatr.

[51] Stanhope R (1988). Preece MA management of constitutional delay of growth and puberty. Arch Dis Child.

[52] Bozzola M, Bozzola E, Montalbano C, Stamati FA, Ferrara P, Villani A (2018). Delayed puberty versus hypogonadism: a challenge for the pediatrician. Ann Pediatr Endocrinol Metab.

[53] Poyrazoglu S, Giinoz H, Darendeliler F, Saka N, Bundak R, Ba F (2005). Constitutional delay of growth and puberty: from presentation to final height. J Pediatr Clin Endocrinol Metab.

[54] Gaudino R, De Filippo G, Bozzola E, Gasparri M, Bozzola M, Villani A (2022). Current clinical management of constitutional delay of growth and puberty. Ital J Pediatr.

[55] Yang Z, Long X, Shen J, Liu D, Dorman JS, Laporte RE (2005). Epidemics of type 1 diabetes in China. Pediatr Diabetes.

[56] Patterson CC, Harjutsalo V, Rosenbauer J, Neu A, Cinek O, Skrivarhaug T (2019). Trends and cyclical variation in the incidence of childhood type 1 diabetes in 26 european centres in the 25 year period 1989–2013: a multicentre prospective registration study. Diabetologia.

[57] Passanisi S, Salzano G, Aloe M, Bombaci B, Citriniti F, De Berardinis F (2022). Increasing trend of type 1 diabetes incidence in the pediatric population of the Calabria region in 2019–2021. Ital J Pediatr.

[58] Barrett CE, Koyama AK, Alvarez P, Chow W, Lundeen EA, Perrine CG (2022). Risk for newly diagnosed diabetes > 30 days after SARS-CoV-2 infection among persons aged < 18 years - United States, March 1, 2020-June 28, 2021. MMWR Morb Mortal Wkly Rep.

[59] Goodman NF, Cobin RH, Futterweit W, Glueck JS, Legro RS, Carmina E, AMERICAN ASSOCIATION OF CLINICAL ENDOCRINOLOGISTS, AMERICAN COLLEGE OF ENDOCRINOLOGY, AND ANDROGEN EXCESS AND PCOS SOCIETY DISEASE STATEClinical review (2015). Guide to the best practice in the evaluation and treatment of policistic ovary Syndrome—Part1. Endocr Pract.

[60] Graham RA, Davis JA, Corrales-Medina FF (2018). The adolescent with menorrhagia: diagnostic approach to a suspected bleeding disorder. Pediatr Rev.

[61] Barbero A, Pagano M, Tuli G, Buganza R, de Sanctis L, Bondone C (2022). Menorrhagia as main presentation sign of severe hypothyroidism in a pediatric patient: a case report. Ital J Pediatr.

[62] Marr A, Hardy K, Curtis J (2018). A 14-year-old girl with short stature, incomplete puberty and severe menstrual bleeding. Paediatr Child Health.

[63] Pulvirenti G, Sortino V, Manti S, Parisi GF, Papale M, Giallongo A (2022). Pathogenesis, diagnosis, dietary management, and prevention of gastrointestinal disorders in the paediatric population. Ital J Pediatr.

[64] Caffarelli C, Di Mauro D, Mastrorilli C, Bottau P, Cipriani F, Ricci G (2018). Solid food introduction and the development of food allergies. Nutrients.

[65] Stallings V, Tindall A, Mascarenhas M, Maqbool M, Schall J. Improved residual fat malabsorption and growth in children with cystic fibrosis treated with a novel oral structured lipid supplement: A randomized controlled trial. PLoS ONE. 2020;15: e0232685. 10.1371/journ.pone.0232685. 10.1371/journal.pone.0232685 PMC7209323 32384122

[66] Hebuterne X, Filippi J, Al-Jaouni R, Schneider S (2009). Nutritional consequences and nutrition therapy in Crohn’s disease. Gastroenterol Clin Biol.

[67] Li BU, Lefevre F, Chelimsky GG, Boles RG, Nelson SP, Lewis DW (2008). North american society for pediatric gastroenterology, hepatology, and nutrition consensus statement on the diagnosis and management of cyclic vomiting syndrome. J Pediatr Gastroenterol Nutr.

[68] Isoldi S, Di Nardo G, Mallardo S, Parisi P, Raucci U, Tambucci R (2022). Cyclic vomiting syndrome in children: a nationwide survey of current practice on behalf of the italian society of Pediatric Gastroenterology, Hepatology and Nutrition (SIGENP) and italian society of Pediatric Neurology (SINP). Ital J Pediatr.

[69] Bhandari S, Jha P, Thakur A, Kar A, Gerdes H, Venkatesan T (2018). Cyclic vomiting syndrome: epidemiology, diagnosis, and treatment. Clin Auton Res.

[70] Li BUK (2018). Managing cyclic vomiting syndrome in children: beyond the guidelines. Eur J Pediatr.

[71] Caffarelli C, Cardinale F, Povesi-Dascola C, Dodi I, Mastrorilli V, Ricci G (2015). Use of probiotics in pediatric infectious diseases. Expert Rev Anti Infect Ther.

[72] Berends MJ, Tan-Sindhunata G, Leegte B, van Essen AJ (2001). Phenotypic variability of Cat-Eye Syndrome. Genet Couns.

[73] Hernández-Medrano C, Hidalgo-Bravo A, Villanueva-Mendoza C, Bautista-Tirado T, Apam-Garduño D (2021). Mosaic cat eye syndrome in a child with unilateral iris coloboma. Ophthalmic Genet.

[74] Serra G, Giambrone C, Antona V, Cardella F, Carta M, Cimador M (2022). Congenital hypopituitarism and multiple midline defects in a newborn with non-familial Cat Eye Syndrome. Ital J Pediatr.

[75] Denavit TM, Malan V, Grillon C, Sanlaville D, Ardalan A, Jacquemont ML (2004). A new case of a severe clinical phenotype of the cat-eye syndrome. Genet Couns.

[76] Shaffer LG, Ledbetter DH, Lupski JR. Molecular cytogenetics of contiguous gene syndromes: Mechanisms and consequences of gene dosage imbalance. In: Valle DL, Antonarakis S, Ballabio A, Beaudet AL, Mitchell GA. eds. The online metabolic and molecular bases of inherited disease. McGraw Hill; 2019. Accessed July 10, 2023. https://ommbid.mhmedical.com/content.aspx?bookid=2709&sectionid=225079178

[77] Serra G, Antona V, Giuffrè M, Piro E, Salerno S, Schierz IAM (2022). Interstitial deletions of chromosome 1p: novel 1p31.3p22.2 microdeletion in a newborn with craniosynostosis, coloboma and cleft palate, and review of the genomic and phenotypic profiles. Ital J Pediatr.

[78] Blessing M, Gallagher ER (2022). Epidemiology, genetics, and pathophysiology of craniosynostosis. Oral Maxillofac Surg Clin North Am.

[79] Fuller ZL, Berg JJ, Mostafavi H, Sella G, Przeworski M (2019). Measuring intolerance to mutation in human genetics. Nat Genet.

[80] Hebron KE, Hernandez ER, Yohe ME (2022). The RASopathies: from pathogenetics to therapeutics. Dis Model Mech.

[81] Serra G, Felice S, Antona V, Di Pace MR, Giuffrè M, Piro E (2022). Cardio-facio-cutaneous syndrome and gastrointestinal defects: report on a newborn with 19p13.3 deletion including the MAP 2 K2 gene. Ital J Pediatr.

[82] Liao Y, Wen H, Ouyang S, Yuan Y, Bi J, Guan Y (2021). Routine first-trimester ultrasound screening using a standardized anatomical protocol. Am J Obstet Gynecol.

[83] Schindewolf E, Moldenhauer JS (2020). Genetic counseling for fetal gastrointestinal anomalies. Curr Opin Obstet Gynecol.

[84] Zampieri N, Pulvirenti R, Pedrazzoli E, Camoglio FS (2022). Hao-Fountain syndrome and genital disorders: report of a new possible association. Ital J Pediatr.

[85] Zheng H, Mei S, Li F, Wei L, Wang Y, Huang J (2022). Expansion of the mutation spectrum and phenotype of USP7-related neurodevelopmental disorder. Front Mol Neurosci.

[86] Priolo M, Mancini C, Pizzi S, Chiriatti L, Radio FC, Cordeddu V (2022). Complex presentation of Hao-Fountain syndrome solved by exome sequencing highlighting co-occurring genomic variants. Genes (Basel).

[87] Saha G, Roy S, Basu M, Ghosh MK (2023). USP7 - a crucial regulator of cancer hallmarks. Biochim Biophys Acta Rev Cancer.

[88] Costantino C, Mazzucco W, Scarpitta F, Ventura G, Marotta C, Bono SE (2022). Prevalence and factors associated with bullying phenomenon among pre-adolescents attending first-grade secondary schools of Palermo, Italy, and a comparative systematic literature review. Ital J Pediatr.

[89] Wiertsema M, Vrijen C, van der Ploeg R, Sentse M, Kretschmer T (2023). Bullying perpetration and social status in the peer group: a meta-analysis. J Adolesc.

[90] Gizzarelli E, Burns S, Francis J (2023). School staff responses to student reports of bullying: a scoping review. Health Promot J Austr.

[91] Chen Q, Chan KL, Guo S, Chen M, Lo CK, Ip P (2023). Effectiveness of digital health interventions in reducing bullying and cyberbullying: a meta-analysis. Trauma Violence Abuse.

[92] Li G (2023). The relationship between mobile phone dependence and subjective well-being of college students in China: a moderated mediation model. Healthc (Basel).

[93] Li Y, Wang Z, You W, Liu X (2022). Core self-evaluation, mental health and mobile phone dependence in chinese high school students: why should we care. Ital J Pediatr.

[94] Bi J (2022). The relationship between mobile phone anxiety and sleep quality occupational therapy in adolescents and its internal mechanism. Occup Ther Int.

[95] Liu H, Novotný JS, Váchová L (2022). The effect of mobile phone addiction on perceived stress and mediating role of ruminations: evidence from chinese and czech university students. Front Psychol.

[96] Offidani C, Villani A, Reale A, Marchili MR, Aufiero LR, Moras P (2022). Early recognition of child abuse through screening indicators at the emergency department: experience of a tertiary urban pediatric hospital. Ital J Pediatr.

[97] Beebe R, Fish MC, Grasso D, Bernstein B, DiVietro S, Stover CS. Reducing family violence through child welfare intervention: a propensity score-matched study of fathers for change. J Interpers Violence. 2023;8862605231186121. 10.1177/08862605231186121. 10.1177/08862605231186121 37470201

[98] Baab SM, Lawsing JF, Macalino CS, Springer JH, Cline DM. Nonaccidental pediatric trauma; which traditional clues predict abuse? Pediatr Emerg Care. 2023 Jul 19. 10.1097/PEC.0000000000003012. Online ahead of print. 10.1097/PEC.0000000000003012 37463155

[99] Mora-Theuer EA, Klomfar S, Ramazanova D, Grylli C, Kletecka-Pulker M, Völkl-Kernstock S (2023). Cohort analysis of child abuse and neglect cases treated during the initial 2 years of a programme to support hospital-based child protection work in Austria. BMJ Open.

[100] Tiruneh CM, Belachew A, Mulatu S, Emiru TD, Tibebu NS, Abate MW (2022). Magnitude of mortality and its associated factors among burn victim children admitted to South Gondar zone government hospitals, Ethiopia, from 2015 to 2019. Ital J Pediatr.

[101] Müller Dittrich MH, Brunow de Carvalho W, Lopes Lavado E (2016). Evaluation of the early use of albumin in children with extensive burns: a randomized controlled trial. Pediatr Crit Care Med.

[102] Canty KW, DeRidder CA (2023). Burns in Children: Accidental or inflicted?. Adv Pediatr.

[103] Halim N, Holland AJA, McMaugh A, Cameron CM, Lystad RP, Badgery-Parker T et al. Impact of childhood burns on academic performance: a matched population-based cohort study. Arch Dis Child 2023 Jul 9:archdischild–2023. 10.1136/archdischild-2023-325769. 10.1136/archdischild-2023-325769 PMC10511986 37423641

[104] UNICEF, Levels, and Trends In Child Malnutrition: UNICEF/WHO/World Bank Group Joint Estimate Malnutrition. ; 2016. p. 1–8. http://data.unicef.org/wp-content/uploads/ Accessed 9 July 2023.

[105] Kuchenbecker J, Reinbott A, Mtimuni B, Krawinkel MB, Jordan I. Nutrition education improves dietary diversity of children 6–23 months at community-level: results from a cluster randomized controlled trial in Malawi. PLoS ONE. 2017;1–19. 10.1371/journal.pone.0175216. 10.1371/journal.pone.0175216 PMC5398527 28426678

[106] Bidira K, Tamiru D, Belachew T (2022). Effect of nutritional education on anthropometric deficits among pre-school aged children in south West Ethiopia: quasi-experimental study. Ital J Pediatr.

[107] Sharma N, Gupta M, Aggarwal AK, Gorle M (2020). Effectiveness of a culturally appropriate nutrition educational intervention delivered through health services to improve growth and complementary feeding of infants: a quasi-experimental study from Chandigarh. India PLoS One.

[108] La Vecchia A, Ippolito G, Taccani V, Gatti E, Bono P, Bettocchi S (2022). Epidemiology and antimicrobial susceptibility of Staphylococcus aureus in children in a tertiary care pediatric hospital in Milan, Italy, 2017–2021. Ital J Pediatr.

[109] Slott Jensen ML, Chen M, Detlefsen M, Kudsk Klitgaard J, Andersen TE, Kemp M (2022). Whole-genome sequence analyses by a new easy-to-use software solution support the suspicion of a neonatal ward outbreak of methicillin-resistant Staphylococcus aureus (MRSA) and transmission between hospitals. Infect Control Hosp Epidemiol.

[110] Quan KA, Sater MRA, Uy C, Clifton-Koeppel R, Dickey LL, Wilson W (2023). Epidemiology and genomics of a slow outbreak of methicillin-resistant Staphyloccus aureus (MRSA) in a neonatal intensive care unit: successful chronic decolonization of MRSA-positive healthcare personnel. Infect Control Hosp Epidemiol.

[111] Lanckohr C, Bracht H (2022). Antimicrobial stewardship. Curr Opin Crit Care.

[112] Accomando S, Restivo GA, Scalzo S, Guardino M, Corsello G, Giuffrè M (2022). Epstein-Barr virus-associated acute pancreatitis: a clinical report and review of literature. Ital J Pediatr.

[113] Ming Y, Cheng S, Chen Z, Su W, Lu S, Wang N (2023). Infectious mononucleosis in children and differences in biomarker levels and other features between disease caused by Epstein-Barr virus and other pathogens: a single-center retrospective study in China. PeerJ.

[114] Capparelli MA, Cotignola L, Domínguez MV, D’Alessandro PD, Ayarzabal VH, Barrenechea ME. Clinical utility of definitive diagnostic tests for choledocholithiasis in pediatric patients with mild gallstone pancreatitis. J Pediatr Surg. 2023;S0022–34682300393. 10.1016/j.jpedsurg.2023.06.011. 10.1016/j.jpedsurg.2023.06.011 37460346

[115] Daniluk U, Krawiec P, Pac-Kożuchowska E, Dembiński Ł, Bukowski JS, Banaszkiewicz A (2023). Pancreatic involvement in the course of inflammatory bowel disease in children-A multi-center study. J Clin Med.

[116] Fahed AC, Nemer GM (2011). Familial hypercholesterolemia: the lipids or the genes?. Nutr Metab.

[117] Banderali G, Capra ME, Biasucci G, Stracquadaino R, Viggiano C, Pederiva C (2022). Detecting familial hypercholesterolemia in children and adolescents: potential and challenges. Ital J Pediatr.

[118] Wiegman A, Gidding SS, Watts GF, Chapman MJ, Ginsberg HN, Cuchel M (2015). Familial hypercholesterolaemia in children and adolescents: gaining decades of life by optimizing detection and treatment. Eur Heart J.

[119] Pederiva C, Capra ME, Viggiano C, Rovelli V, Banderali G, Biasucci G (2021). Early prevention of atherosclerosis: detection and management of hypercholesterolaemia in children and adolescents. Life.

[120] Gragnaniello V, Deodato F, Gasperini S, Donati MA, Canessa C, Fecarotta S (2022). Immune responses to alglucosidase in infantile pompe disease: recommendations from an italian pediatric expert panel. Ital J Pediatr.

[121] Ditters IAM, van der Beek NAME, Brusse E, van der Ploeg AT, van den Hout JMP, Huidekoper HH (2023). Home-based enzyme replacement therapy in children and adults with pompe disease; a prospective study. Orphanet J Rare Dis.

[122] Kishnani PS, Kronn D, Brassier A, Broomfield A, Davison J, Hahn SH (2023). Safety and efficacy of avalglucosidase alfa in individuals with infantile-onset pompe disease enrolled in the phase 2, open-label Mini-COMET study: the 6-month primary analysis report. Genet Med.

[123] Ertoy Karagol HI, Inci A, Terece SP, Kilic A, Demir F, Yapar D (2023). Long-term experience with anaphylaxis and desensitization to alglucosidase alfa in pompe disease. Int Arch Allergy Immunol.

[124] Stein Duker LI, Kwon J, Richter M, Pineda R (2023). Feasibility of wearable sensors in the NICU: psychophysiological measures of parental stress. Early Hum Dev.

[125] Orovou E, Eskitzis P, Mrvoljak-Theodoropoulou I, Tzitiridou-Hatzopoulou M, Dagla M, Arampatzi C (2023). The relation between neonatal intensive care units and postpartum post-traumatic stress disorder after cesarean section. Healthc (Basel).

[126] Salomè S, Mansi G, Lambiase CV, Barone M, Piro V, Pesce M (2022). Impact of psychological distress and psychophysical wellbeing on posttraumatic symptoms in parents of preterm infants after NICU discharge. Ital J Pediatr.

[127] Mielewczyk FJ, Boyle EM (2023). Uncharted territory: a narrative review of parental involvement in decision-making about late preterm and early term delivery. BMC Pregnancy Childbirth.

[128] Baldassarre ME, Panza R, Cresi F, Salvatori G, Corvaglia L, Aceti A (2022). Complementary feeding in preterm infants: a position paper by italian neonatal, paediatric and paediatric gastroenterology joint societies. Ital J Pediatr.

[129] Haiden N, Haschke F (2023). Early nutrition must be safe and should have positive impacts on long-term health. Nutrients.

[130] Gsoellpointner M, Eibensteiner F, Thanhaeuser M, Ristl R, Jilma B, Berger A (2023). Effects of early introduction of solid foods on nutrient intake in preterm infants during their 1st year of life: a secondary outcome analysis of a prospective, randomized intervention study. Front Nutr.

[131] Giannì ML, Consales A, Morniroli D, Vizzari G, Mosca F (2023). The Breastfeeding Paradox as a guide for the assessment of premature infants growth: it is more than just weigh-ins. Breastfeed Med.

[132] Alemu A, Molla W, Yinges K, Mihret MS (2022). Determinants of HIV infection among children born to HIV positive mothers on prevention of mother to child transmission program at referral hospitals in west Amhara, Ethiopia; case control study. Ital J Pediatr.

[133] Fassinou LC, Hien H, Yombi JC, Nagot N, Kirakoya-Samadoulougou F (2023). Availability and readiness of the health facilities to provide HIV counseling and testing and prevention of mother-to-child transmission services in Burkina Faso: a trend analysis from 2012 to 2018. BMC Health Serv Res.

[134] Bell L, van Gemert C, Allard N, Brink A, Chan PL, Cowie B (2023). Progress towards triple elimination of mother-to-child transmission of HIV, hepatitis B and syphilis in Pacific Island Countries and Territories: a systematic review. Lancet Reg Health West Pac.

[135] Bahri N, Khaksariyan Z, Khajavian N, Mohammadzadeh A (2023). Educational needs assessment of medical and midwifery students about prevention of mother-to-child transmission of HIV: a cross-sectional study. Virusdisease.

[136] Perez Y, Qureshi MY, Babovic-Vuksanovic D, Cannon B (2023). Aortic dissection in a young patient with unsuspected aortopathy. Pediatrics.

[137] Barton M, Wang H (2023). An uncommon presentation of acute thoracic aortic dissection. J Clin Med Res.

[138] Baldo F, Morra L, Feresin A, Faletra F, Al Naber Y, Memo L (2022). Neonatal presentation of Loeys-Dietz syndrome: two case reports and review of the literature. Ital J Pediatr.

[139] Nakajima T, Iba Y, Shibata T, Hosaka I, Nakazawa J, Kawaharada N (2023). Ten-year follow-up study of a young woman with loeys-dietz syndrome: a case report. J Cardiothorac Surg.

[140] Lazarus G, Francie J, Roeslani RD, Saldi SRF, Oswari H (2022). Role of ursodeoxycholic acid in neonatal indirect hyperbilirubinemia: a systematic review and meta-analysis of randomized controlled trials. Ital J Pediatr.

[141] Vakili O, Mafi A, Pourfarzam M. Liver disorders caused by inborn errors of metabolism. Endocr Metab Immune Disord Drug Targets. 2023 Jun 23. 10.2174/1871530323666230623120935. Online ahead of print. 10.2174/1871530323666230623120935 37357514

[142] Wang YS, Shen W, Yang Q, Lin R, Tang LX, Bai RM (2023). Analysis of risk factors for parenteral nutrition-associated cholestasis in preterm infants: a multicenter observational study. BMC Pediatr.

[143] Babaie E, Hassanpour K, Aldaghi M, Sahebkar M (2023). Comparison of the effect of ursodeoxycholic acid and multistrain synbiotic on indirect hyperbilirubinemia among neonates treated with phototherapy: a double-blind, randomized, placebo-controlled clinical trial study. J Res Med Sci.

[144] Cozzi L, Nuti F, Degrassi I, Civeriati D, Paolella G, Nebbia G (2022). Gilbert or Crigler-Najjar syndrome? Neonatal severe unconjugated hyperbilirubinemia with P364L UGT1A1 homozygosity. Ital J Pediatr.

[145] Germana S, Shaikh SK (2023). Increasing utilisation of a rebound hyperbilirubinaemia calculator in two newborn nurseries. BMJ Open Qual.

[146] Espinosa K, Brown SR (2023). Hyperbilirubinemia in newborns: updated guidelines from the AAP. Am Fam Physician.

[147] Kasirer Y, Kaplan M, Hammerman C (2023). Kernicterus on the spectrum. Neoreviews.

[148] Dell’Olio F, Baldassarre ME, Russo FG, Schettini F, Siciliani RA, Mezzapesa PP (2022). Lingual laser frenotomy in newborns with ankyloglossia: a prospective cohort study. Ital J Pediatr.

[149] Cherian D, Saeed R, Anusha K, Rag B, Peter T (2023). Management of ankyloglossia by functional frenuloplasty using diode laser. J Orthod Sci.

[150] Borowitz SM (2023). What is tongue-tie and does it interfere with breast-feeding? - a brief review. Front Pediatr.

[151] Akbari D, Bogaardt H, Docking K (2023). Ankyloglossia in Central Australia: prevalence, identification and management in infants. Int J Pediatr Otorhinolaryngol.

[152] Hu Y, Chen F, Xiang X, Wang F, Hua Z, Wei H (2022). Early versus delayed enteral nutrition for neonatal hypoxic-ischemic encephalopathy undergoing therapeutic hypothermia: a randomized controlled trial. Ital J Pediatr.

[153] Pandya F, Mukherji A, Goswami I. An exploratory analysis of gastrointestinal morbidities and feeding outcomes associated with neonatal hypoxic-ischemic encephalopathy with or without hypothermia therapy. Ther Hypothermia Temp Manag. 2023 May;3. 10.1089/ther.2023.0008. 10.1089/ther.2023.0008 37140459

[154] Kumar J, Anne RP, Meena J, Sundaram V, Dutta S, Kumar P (2023). To feed or not to feed during therapeutic hypothermia in asphyxiated neonates: a systematic review and meta-analysis. Eur J Pediatr.

[155] Sharma S, Kallesh A, Aradhya AS, Diggikar S, Veeraiah PS, Subbareddy NN (2023). Feasibility of minimal enteral nutrition during therapeutic hypothermia for perinatal asphyxia: a five-year multicenter experience from South India. Indian J Pediatr.

[156] Eiland LS (2012). Glycopyrrolate for chronic drooling in children. Clin Ther.

[157] Walshe M, Smith M, Pennington L (2012). Interventions for drooling in children with cerebral palsy. Cochrane Database Syst Rev.

[158] Blasco PA, Stansbury JC (1996). Glycopyrrolate treatment of chronic drooling. Arch Pediatr Adolesc Med.

[159] Lovardi E, De Ioris MA, Lettori D, Geremia C, Staccioli S, Bella GD (2022). Glycopyrrolate for drooling in children with medical complexity under three years of age. Ital J Pediatr.

[160] Verret S, Steele JC (1971). Alternating hemiplegia in childhood: a report of eight patients with complicated migraine beginning in infancy. Pediatrics.

[161] Heinzen EL, Swoboda KJ, Hitomi Y, Gurrieri F, Nicole S, de Vries B (2012). De novo mutations in ATP1A3 cause alternating hemiplegia of childhood. Nat Genet.

[162] Pavone P, Pappalardo XG, Mustafa N, Cho SY, Jin DK, Incorpora G (2022). Alternating hemiplegia of childhood: neurological comorbidities and intrafamilial variability. Ital J Pediatr.

[163] Mikati MA, Kramer U, Zupanc ML, Shanahan RJ (2000). Alternating hemiplegia of childhood: clinical manifestations and long-term outcome. Pediatr Neurol.

[164] Association A, American Psychiatric Association (2013). Diagnostic and statistical manual of mental disorders: DSM-5.

[165] Lord C, Elsabbagh M, Baird G, Veenstra-Vanderweele J (2018). Autism spectrum disorder. Lancet.

[166] Nevison CD, Blaxill M (2017). Diagnostic substitution for intellectual disability: a flawed explanation for the rise in autism. J Autism Dev Disord.

[167] Salari N, Rasoulpoor S, Rasoulpoor S, Shohaimi S, Jafarpour S, Abdoli N (2022). The global prevalence of autism spectrum disorder: a comprehensive systematic review and meta-analysis. Ital J Pediatr.

[168] Hyman SL, Levy SE, Myers SM (2020). Council on children with disabilities, section on developmental and behavioral pediatrics identification, evaluation, and management of children with autism spectrum disorder. Pediatr.

[169] Barth PG (1993). Pontocerebellar hypoplasias: an overview of a group of inherited neurodegenerative disorders with fetal onset. Brain Dev.

[170] Kasinathan A, Sankhyan N, Dijk TV, Singh P, Singh P (2020). Clinico-radiological profile of children with pontocerebellar hypoplasia. J Pediatr Neurosce.

[171] Bilge S, Mert GG, Hergüner Ö, Özcanyüz D, Bozdoğan ST, Kaya Ö (2022). Clinical, radiological, and genetic variation in pontocerebellar hypoplasia disorder and our clinical experience. Ital J Pediatr.

[172] D’Agostino R, Tarantino V, Melagrana A, Taborelli G (1997). Otoneurologic evaluation of child vertigo. Int J Pediatr Otorhinolaryngol.

[173] Gruber M, Cohen-Kerem R, Kaminer M, Shupak A (2012). Vertigo in children and adolescents: characteristics and outcome. ScientificWorldJournal.

[174] Pellegrino N, Di Stefano V, Rotondo E, Graziosi A, Rispoli MG, Torrente A (2022). Neurological vertigo in the emergency room in pediatric and adult age: systematic literature review and proposal for a diagnostic algorithm. Ital J Pediatr.

[175] Raucci U, Vanacore N, Paolino MC, Silenzi R, Mariani R (2015). Vertigo/dizziness in pediatric emergency department: five years’ experience. Cephalalgia.

[176] Del Monte F, Bucchino L, Versace A, Tardivo I, Castagno E, Pieri G (2022). Infantile idiopathic intracranial hypertension: case report and review of the literature. Ital J Pediatr.

[177] Gaier ED, Heidary G (2019). Pediatric idiopathic intracranial hypertension. Semin Neurol.

[178] Gillson N, Jones C, Reem RE, Rogers DL, Zumberge N, Aylward SC (2017). Incidence and demographics of pediatric intracranial hypertension. Pediatr Neurol.

[179] Ravid S, Shahar E, Schif A, Yehudian S (2015). Visual outcome and recurrence rate in children with idiopathic intracranial hypertension. J Child Neurol.

[180] Karklinsky S, Kugler S, Bar-Yosef O, Nissenkorn A, Grossman-Jonish A, Tirosh I (2022). Hereditary neuropathy with liability to pressure palsies (HNPP): intrafamilial phenotypic variability and early childhood refusal to walk as the presenting symptom. Ital J Pediatr.

[181] Choi HW, Kuntz NL (2015). Hereditary neuropathy with liability to pressure palsies. Pediatr Neurol Briefs.

[182] Chrestian N, McMillan H, Poulin C, Campbell C, Vajsar J (2015). Hereditary neuropathy with liability to pressure palsies in childhood: case series and literature update. Neuromuscul Disord.

[183] Felice KJ, Leicher CR, DiMario FJ (1999). Hereditary neuropathy with liability to pressure palsies in children. Pediatr Neurol.

[184] Pavone P, Corsello G, Raucci U, Lubrano R, Parano E, Ruggieri M (2022). Febrile infection-related Epilepsy Syndrome (FIRES): a severe encephalopathy with status epilepticus. Literature review and presentation of two new cases. Ital J Pediatr.

[185] Rachfalska N, Pietruszewski J, Paprocka J (2021). Dramatic course of paediatric cryptogenic febrile infection-related epilepsy syndrome with unusual chronic phase presentation-A case report with literature study. Brain Sci.

[186] Lee Y-J (2020). Febrile infection-related epilepsy syndrome: refractory status epilepticus and management strategies. Ann Child Neurol.

[187] Sheikh Z, Hirsch LJ (2023). A practical approach to in-hospital management of new-onset refractory status epilepticus/febrile infection related epilepsy syndrome. Front Neurol.

[188] Kramer U, Chi CS, Lin KL, Specchio N, Sahin M, Olson H (2011). Febrile infection-related epilepsy syndrome (FIRES): pathogenesis, treatment, and outcome: a multicenter study on 77 children. Epilepsia.

[189] Helbig I (2015). Genetic causes of generalized epilepsies. Semin Neurol.

[190] Ghazala E, Shahin DA, Wahba Y (2022). Polymorphisms of the sodium voltage-gated channel, alpha subunit 1 (SCN1A -A3184G) gene among children with non-lesional epilepsy: a case-control study. Ital J Pediatr.

[191] Hille B (2001). Ion channels of excitable membranes.

[192] Baum L, Haerian BS, Ng HK, Wong VC, Ng PW, Lui CH (2014). Case–control association study of polymorphisms in the voltage-gated sodium channel genes SCN1A, SCN2A, SCN3A, SCN1B, and SCN2B and epilepsy. Hum Genet.

[193] Trapani S, Bortone B, Bianconi M, Rubino C, Sardi I, Lionetti P (2022). Diencephalic syndrome in childhood, a challenging cause of failure to thrive: miniseries and literature review. Ital J Pediatr.

[194] Murphy AM, Drumm B, Brenner C, Lynch SA (2006). Diencephalic cachexia of infancy: Russell’s syndrome. Clin Dysmorphol.

[195] Satyarthee GD, Chipde H (2018). Diencephalic syndrome as presentation of giant childhood craniopharyngioma: a management review. J Pediatr Neurosci.

[196] Sardi I, Bresci C, Schiavello E, Biassoni V, Fratoni V, Cardellicchio S (2012). Successful treatment with a low-dose cisplatin–etoposide regimen for patients with diencephalic syndrome. J Neurooncol.

[197] Steliarova-Foucher E, Colombet M, Ries LAG, Moreno F, Dolya A, Bray F (2017). International incidence of childhood cancer, 2001-10: a population-based registry study. Lancet Oncol.

[198] Lim HA, Tan JY, Chua J, Yoong RK, Lim SE, Kua EH (2017). Quality of life of family caregivers of cancer patients in Singapore and globally. Singap Med J.

[199] Chaghazardi M, Janatolmakan M, Rezaeian S, Khatony A (2022). Care burden and associated factors in caregivers of children with cancer. Ital J Pediatr.

[200] Jafari H, Ebrahimi A, Aghaei A, Khatony A (2018). The relationship between care burden and quality of life in caregivers of hemodialysis patients. BMC Nephrol.

[201] Virgili F, Nenna R, Ben David S, Mancino E, Di Mattia G, Matera L (2022). E-cigarettes and youth: an unresolved Public Health concern. Ital J Pediatr.

[202] CDC. About Electronic Cigarettes. https://www.cdc.gov/tobacco/basic_information/e-cigarettes/about-e-cigarettes.html. Accessed 9 Apr 2021.

[203] Cherian SV, Kumar A, Estrada-Y-Martin RM (2020). E-Cigarette or vaping product-associated lung injury: a review. Am J Med.

[204] Layden JE, Ghinai I, Pray I, Kimball A, Layer M, Tenforde MW (2020). Pulmonary illness related to E-cigarette use in Illinois and Wisconsin - Final Report. N Engl J Med.

[205] ISTAT. https://www.salute.gov.it/portale/fumo/dettaglio. Accessed 26 June 2022.

[206] Casamento Tumeo C, Schiavino A, Paglietti MG, Petreschi F, Ottavianelli A, Onofri A (2022). E-cigarette or vaping product use Associated Lung Injury (EVALI) in a 15 year old female patient - case report. Ital J Pediatr.

[207] Siegel DA, Jatlaoui TC, Koumans EH, Kiernan EA, Layer M, Cates JE (2019). Update: interim guidance for health care providers evaluating and caring for patients with suspected e-cigarette or vaping, product use associated lung injury – United States, October 2019. Morb Mortal Wkly Rep.

[208] Layden JE, Ghinai I, Pray I, Kimball A, Layer M, Tenforde MW (2020). Pulmonary illness related to E-cigarette use in Illinois and Wisconsin - Final Report. N Engl J Med.

[209] Blount BC, Karwowski MP, Shields PG, Morel-Espinosa M, Valentin-Blasini L, Gardner M (2020). Vitamin E acetate in bronchoalveolar-lavage fluid associated with EVALI. N Engl J Med.

[210] Bell SC, Mall MA, Gutierrez H, Macek M, Madge S, Davies JC (2020). The future of cystic fibrosis care: a global perspective. Lancet Respir Med.

[211] Cabral B, Terlizzi V, Laselva O, Conte Filho C, Mota F (2022). Anticipating new treatments for cystic fibrosis: a global survey of researchers. J Clin Med.

[212] Terlizzi V, Castellani C, Taccetti G, Ferrari B (2022). Dornase alfa in cystic fibrosis: indications, comparative studies and effects on lung clearance index. Ital J Pediatr.

[213] Castellani C, Duff AJA, Bell SC, Heijerman HGM, Munck A, Ratjen F (2018). ECFS best practice guidelines: the 2018 revision. J Cyst Fibros.

